# Genetic engineering of low-temperature polyhydroxyalkanoate production by *Acidovorax* sp. A1169, a psychrophile isolated from a subglacial outflow

**DOI:** 10.1007/s00792-023-01311-5

**Published:** 2023-09-14

**Authors:** Jakub Grzesiak, Jan Gawor, Małgorzata Marta Rogala, Xenie Kouřilová, Stanislav Obruča

**Affiliations:** 1 Institute of Biochemistry and Biophysics, Polish Academy of Sciences, Pawińskiego 5A, 02-106 Warsaw, Poland; 2 Department of Food Chemistry and Biotechnology, Faculty of Chemistry, Brno University of Technology, Purkynova 118, 612 00 Brno, Czech Republic

**Keywords:** Extremophile, Arctic, Bioplastics, Low-temperature biotechnology, Oligotrophy

## Abstract

In recent years, extremophilic microorganisms have been employed as producers of the microbial bioplastics polyhydroxyalkanoates (PHA), which are of great biotechnological value. Nevertheless, cold-loving or psychrophilic (cryophilic) bacteria have been neglected in this regard. Here, we present an investigation of the Arctic glacier-derived PHA producer *Acidovorax* sp. A1169. Biolog GEN III Microplates were used as a screening tool to identify the most suitable carbon substrate concerning PHA synthesis. The strain produced homopolymer poly(3-hydroxybutyrate) (PHB) most efficiently (2 g/L) at a temperature of 15 °C when supplied with fructose or mannitol as carbon sources with a substantial decrease of PHB biosynthesis at 17.5 °C. The PHB yield did not increase considerably or even decreased when carbon source concentration exceeded 10 g/L hinting that the strain is oligotrophic in nature. The strain was also capable of introducing 3-hydroxyvalerate (3HV) into the polymer structure, which is known to improve PHA thermoplastic properties. This is the first investigation providing insight into a PHA biosynthesis process by means of a true psychrophile, offering guidelines on polar-region bacteria cultivation, production of PHA and also on the methodology for genetic engineering of psychrophiles.

## Introduction

Bacterial intracellular storage materials provide its producers with an immense advantage in their native habitats by enabling proliferation at nutrient-deficit circumstances and thus allowing the out competition of species lacking this ability (Moradali and Rehm 2020). One such compound are polyhydroxyalkanoates (PHA), microbial polyesters of hydroxyacids that are stored intracellularly as insoluble granules when carbon source is present in excess to be subsequently depolymerized and catabolized under carbon source limitation conditions. Enzymes responsible for the polymerization process are the PHA synthases encoded by the *phaC* gene. So far, four classes of this enzyme were discovered and described. PHAs can be divided into short-chain length (scl-PHA, C3-C5 per monomer) PHAs and medium-chain length (mcl-PHA, C6-C14) PHA (Koller 2018). Generally, scl-PHA are produced with the participation of enzymes encoded by *phaA* and *phaB* genes and by the action of class I, III and IV PHA synthases, mcl-PHA are synthesized by class II synthases with precursors being delivered by lipid de-novo synthesis or catabolism of long-chain fatty acids (Reddy et al. 2003). The chemical and physical properties of the resulting polymers were what drew the attention of the industry as they resemble those of plastics made from petroleum or its derivatives. Biodegradability and biocompatibility were the defining factors that led to the large-scale production of the PHA polymer (Tan et al. 2021). The most basic and common member of the PHA family is a homopolymer poly(3-hydroxybutyrate) (PHB) produced by Bacteria and Archaea alike (Müller-Santos et al. 2021). Its thermoplastic properties are greatly improved when various copolymers are produced, for instance by the introduction of hydroxyacid with > 4 carbon atoms (such as 3-hydroxyvalerate) (Tan et al. 2021).

Being totally biologically produced, PHA synthesis costs far exceed that of oil-based plastics, so its application is now largely restricted to high-value applications e.g. in the medical field (implants, drug carriers). Therefore, lowering the cost of PHA production is key to increasing PHA competitiveness in the global market (Możejko-Ciesielska and Kiewisz 2016). Several factors of the production process can be optimized in order to make PHA more affordable. Choosing the right PHA producer strain is of pivotal importance. Many strategies involving selection in permissive conditions were applied, leading to the isolation of bacterial and archaeal species with a promising PHA-producing potential. Secondly, the identification of the appropriate carbon source is usually one of the first steps in PHA biosynthesis optimization. Other conditions such as medium composition and pH, and incubation temperature, are also subjects of optimization (Tripathi et al. 2013). A plethora of environmental strains was tested in such a manner, with two being frequently used in science and industry: *Cupriavidus* *necator* (scl-PHA producer) and *Pseudomonas* *putida* (mcl-PHA producer) (Zhang et al. 2022; Weimer et al. 2020). Genetic manipulation can be employed to further increase PHA yields in model producer strains, mainly by enhancing carbon flow into the PHA biosynthesis pathways or preventing intracellular PHA degradation by inactivating PHA depolymerases encoded by the *phaZ* gene (Wang et al. 2023).

In recent years, several “outside the box” microbes have been tested for their ability and efficiency to produce PHAs (Koller 2017). Thermophiles, such as *Schlegelella* *thermodepolymerans* or *Aneurinibacillus* *sp.* as well as halophiles (*Halomonas* spp*.*) were employed to convert waste products into PHAs of varying kinds, with the advantage of unusual cultivation conditions providing the needed robustness of the process against contamination by common mesophilic microflora which substantially reduces the cost associated with sterility demands and opens avenues for establishment of effective continuous or semicontinuous cultivation scenarios (Kourilova et al. 2020; Rehakova et al. 2023; Wang et al. 2022). Therefore, the employment of extremophiles may grant the industry new, surprising benefits as was recently formulated in the concept of Next-Generation Industrial Biotechnology by professor Chen (Chen and Jiang 2018).

Nevertheless, psychrophiles have been pretty much neglected in this respect. They grow best below the temperatures usually applied in industrial microbial biotechnology (*T* < 20 °C) (Madigan et al. 2019). Psychrophiles display several advantages when cultured at low temperatures, *inter* *alia*: decreased energy input, especially practical in colder climates or around the winter season (Margesin et al. 2008). Low temperature also has a stabilizing effect on the substrate and the products of bacterial metabolism, especially important if one of those is thermolabile, like many sugars (Wolfenden and Yuan 2008). Furthermore, the solubility of atmospheric oxygen is greatly improved in lower temperatures, which substantially prevents problems with the aeration of the process and reduces associated costs (Georlette et al. 2004). Psychrophiles are widespread, occupying niches and habitats across the globe, mostly polar and alpine environments as well as the vast expanses of the deep ocean, providing a great opportunity for bioprospecting for PHA producers (Margesin et al. 2008; Rogala et al. 2020). Numerous study indicates that PHA production ability is common among microbes adapted to cold environments (Goh and Tan 2012; Ciesielski et al. 2014; Kumar et al. 2018; Rogala et al. 2020), there are also indications that PHA prevents bacterial cells from the harmful effect of low temperature (Nowroth et al. 2016) and even repeated freezing and thawing (Obruca et al. 2016). Therefore, there were some attempts to employ polar-region bacteria as PHA producers, yet the optimal cultivation temperatures were outside the range of what can be considered psychrophilic, so true low-temperature PHA-production analysis was not published to date to the best of our knowledge (Kumar et al. 2020; Pacheco et al. 2019; Choi et al. 2021).

The aim of the presented research was therefore to explore the capacity of a psychrophilic PHA producer to biosynthesize the polymer at low temperatures and to assess the dynamics of PHA production during submerged cultivation. After an elaborate screening of 200 polar-region bacterial isolates described in Rogala et al. (2020), a chosen few were provisionally analysed by gas chromatography for their PHA content and quality. *Acidovorax* sp. A1169 emerged as one of the more promising strains. It was isolated from the waters of a subglacial outflow stream emerging from beneth Hans Glacier, a tidewater glacier located at the shore of Hornsund fjord at Spitsbergen Island in the Arctic. We hypothesized that *Acidovorax* sp. A1169 could be an efficient PHA producer and given the right conditions it can accumulate PHAs at least to 50% of cell dry weight.

## Materials and methods

### Bacterial strains, plasmids and culture conditions

The bacterial strains and plasmids used in this study are listed in Table 1. The Arctic isolate *Acidovorax* sp. A1169 was obtained from the Central Collection of Strains of the Institute of Biochemistry and Biophysics, Polish Academy of Sciences. It was previously recognized as a potent, low-temperature PHA producer (Rogala et al. 2020). *Escherichia* *coli* strain DH10B was used for plasmid transformation and propagation, while *E*. *coli* strain S17-1 was used for mobilization of the suicide plasmid pAKE604 into *Acidovorax* sp. A1169. Wild-type of *Acidovorax* sp. A1169 strain and its gene knockout mutant (Δ*i-phaZ*) were cultured in R3A medium (1 g/L tryptone, 1 g/L peptone, 1 g/L beef extract, 1 g/L yeast extract, 1 g/L K_2_HPO_4_, 0.5 g/L NaH_2_PO_4_, 0.5 g/L Na-pyruvate, 0.1 g/L MgSO_4_‧7H_2_O) at 15 °C (if not otherwise indicated). All *E.* *coli* strains were cultured in LB broth on a shaker at 200 rpm and 37 °C or on LB agar at 37 °C. Where required, kanamycin was added to a final concentration of 25 or 50 µg/L to ensure plasmid maintenance and selection.

**Table 1:** Bacterial strains and plasmids used in this study

Strain/plasmid	Genotype/phenotype	Source/references
*Acidovorax* sp. A1169		
Wild-type	PHB producer	Arctic glacier/Rogala et al. (2020)
Δ*i-phaZ*	*i-phaZ* gene knockout mutant derived from A1169	This study
*E.* *coli*		
DH10B	F– mcrA Δ(mrr-hsdRMS-mcrBC) φ80lacZΔM15 ΔlacX74 recA1 endA1 araD139 Δ(ara-leu)7697 galU galK λ– rpsL(StrR) nupG	Thermo-Fisher Scientific
S17-1	recA pro hsdR RP4-2-Tc::Mu-Km::Tn7,λ-pir; mobilizer strain	Lab stock
Plasmid pAKE604	ori_MB1_ ori_TRK2_ Ap^r^ Km^r^ lacZ sacB	El-Sayed et al. (2001)

### DNA isolation and sequencing

Genomic DNA was isolated by the CTAB method (Wilson 2001). Plasmid isolation was performed with the Plasmid Midi AX or the Plasmid Mini kits (A&A Biotechnology) while DNA purification was conducted with the Clean-up Concentrator kit (A&A Biotechnology) according to manufacturer instructions. The genome of *Acidovorax* sp. A1169 was sequenced using an Illumina MiSeq apparatus (Illumina Inc., USA). The Illumina paired-end sequencing library construction was performed with 1 μg of post-nebulized DNA extract and the KAPA Library Preparation Kit reagents (KAPA Biosystems, USA), according to the manufacturer’s instructions. The library was pooled and sequenced on a MiSeq platform using the 600-cycle MiSeq reagent Kit v.3 (Illumina, USA). Sequence quality metrics were assessed using FASTQC (Andrews 2010).

### Genome assembly, annotation, primer design, PCR amplification and cloning

Raw sequencing reads were trimmed for quality and residual library adaptors were removed using fastp software (Chen et al. 2018, https://academic.oup.com/bioinformatics/article/34/17/i884/5093234). Cleaned Illumina reads were assembled into contigs using SPAdes software (https://github.com/ablab/spades). Draft genome was annotated using the BV-BRC platform (https://www.bv-brc.org/). The SnapGene program SnapGene software (www.snapgene.com) was used to design primers for the amplification of i-phaZ flanking regions (Table 2). PCR amplifications were performed using PCR Mix Plus, PCR Mix Plus HGC and PCR Mix RAPID ready-to-use mixes for PCR (A&A Biotechnology). Appropriate flanking region pairs were cloned into pAKE604 (Km^r^) vectors using the Anza Restriction Enzyme Cloning System (ThermoFisher) according to the manufacturer’s instructions and then transformed into *E*. *coli* DH10B chemically competent cells made using the Inoue method (Inoue et al. 1990). Transformants were checked by colony PCR using specific primers (Table 2).

**Table 2:** DNA primers used in this study

Primer	Primer sequence (5′–3′)	Note
1169Z1F 1169Z1R	TCAGGGATCCATAACGGAGTTTCGACCCCATGCT (BamHI) TCAGGAATTCGTATCGTCCGAAAAGCGCTTGAA (EcoRI)	For amplifying the 333-bp upstream The homologous sequence of *i-phaZ*
1169Z2F 1169Z2R	TCAGGAATTCCGGCATCTTCAGCGGCCGGCGCTG (EcoRI) TCAGAAGCTTGTGCGGCGGGTGCGTGGTGCCGG (HindIII)	For amplifying the 414-bp downstream The homologous sequence of *i-phaZ*
1169Z1F 1169rZ	TCAGGGATCCATAACGGAGTTTCGACCCCATGCT TTCACCACCGGTTTGCTGGCGA	For the confirmation of the Δ*i-phaZ* knock-out mutant

### Bacterial conjugation

Recombinant plasmids were introduced into *E.* *coli* strain S17-1. Biparental mating with psychrophilic PHB producers was done as follows: saturated cultures of *Acidovorax* sp. A1169 and *E.* *coli* S17-1 were washed with PBS and combined in a 3:1 ratio. The resulting suspension was drop plated onto Conjugation Agar containing: 1 g/L tryptone, 1 g/L peptone, 1 g/L beef extract, 1 g/L yeast extract, 1 g/L K_2_HPO_4_, 0.5 g/L NaH_2_PO_4_, 0.5 g/L Na-pyruvate, 0.1 g/L MgSO_4_‧7H_2_O, 3 g/L HEPES, 3 g/L NORIT^®^ activated charcoal, 15 g/L agar, pH was adjusted to 7.2 with 0.1 M KOH, 0.1 M HCl and a Hanna pH-meter. Plates were incubated at 15 °C for 48 h, after which the growth was scraped, serially diluted and plated onto R3A plates with Kanamycin (25 mg/L) and incubated at 10 °C until single colonies developed. Low temperature was used as a selecting factor for psychrophilic transconjugants as using the traditional method of generating *Acidovorax* sp. A1169 Rifampin resistant mutants, was unsuccessful (Smorawińska et al*.* 2012). Colonies of Km-resistant psychrophiles were picked, inoculated into R3A broth supplemented with 2.5% sucrose and incubated at 10 °C with shaking until bacterial growth was apparent. The resulting suspension was diluted and plated onto R3A plates with 2.5% sucrose and incubated at 15 °C until colony development. Obtained isolates were screened for the target sequence by PCR, using appropriate primers (Table 2).

### Microarray metabolic fingerprinting

Carbon source utilization abilities of *Acidovorax* sp. A1169 were assessed using GEN III Microplates (Biolog Inc., Hayward, CA, USA) as described in Gawor et al. (2016). After incubation in R3A broth on a rotary shaker (WL-972, JWElectronics) for 3 days in 15 °C the cells were harvested by centrifugation (9000 rpm for 3 min), washed twice, suspended in sterile 0.9% saline and added to a vial of MicroPlate IF C inoculation fluid until transmittance reached 90%. Biolog GEN III microplates (Biolog Inc., Hayward, CA, USA) were inoculated according to the manufacturer’s instructions. The plates were incubated in darkness at 15 °C, the color development was read at 590 nm (A590) in a Varioskan plate reader (Thermo Fisher Scientific, Waltham, MA, USA), and cellular respiration was measured kinetically by determining the colorimetric reduction of tetrazolium dye. Data were collected twice a week over a 15-day period.

### PHA production

To assess which carbon source facilitates the most efficient granule synthesis in *Acidovorax* sp. A1169 several compounds, indicated by the Biolog system, were used as substrates for PHA production. PHA synthesis induction was done according to Kourilova et al. (2021a) with modifications. An active inoculum of wild-type strain was prepared (R3A broth, 15 °C, 72 h) and added (10% v/v) to the PHA production medium. The PHA production medium consisted of the following: 9 g/L Na_2_HPO_4_·12H_2_O, 1.5 g/L KH_2_PO_4_, 1 g/L NH_4_Cl, 0.5 g/L yeast extract, 0.2 g/L MgSO_4_·7H_2_O, 0.02 g/L CaCl_2_·2H_2_O and 1 ml/L SL-11 trace element solution (5.2 g/L Na_2_-EDTA, 1.5 g/L FeCl_2_·4H_2_O, 190.0 g/L CoCl_2_·6H_2_O, 100.0 mg/L MnCl_2_·4H_2_O, 70.0 mg/L ZnCl_2_, 36.0 mg/L Na_2_MoO_4_·H_2_O, 24.0 mg/L NiCl_2_·6H_2_O, 6.0 mg/L H_3_BO_3_, 2.0 mg/L CuCl_2_·2H_2_O). After autoclaving a filter-sterilized solution of one of the following substrates was added to each flask of medium to a final conc. of 10 g/L: mannitol, fructose, glycerol, glucose, lactate, sorbitol and mannose. After 96 h of incubation at 15 °C and 150 rpm (INNOVA44 Incubator Shaker, New Brunswick Scientific), the biomass was harvested by centrifugation for further analysis. PHA production efficiency was further investigated at different temperatures (10.0, 12.5, 15.0 and 17.5 °C) with fructose and mannitol as carbon sources (10 g/L) after 96 h at 15 °C and 150 rpm. To assess the optimum duration of the incubation period biomass and PHB content were measured daily for 6 days at 15 °C and 150 rpm. The nitrogen source effect on the PHB accumulation was measured after substituting the NH_4_Cl in the production medium with urea (0.56 g/L) or peptone (1.73 g/L). The nitrogen source concentration was calculated to equate to that of 1 g/L of NH_4_Cl assuming the peptone nitrogen content was 15%. The carbon source concentration effect was investigated by adding mannitol or fructose to the PHA production medium at a conc. of 10 and 20 g/L. The strain’s ability of 3HV incorporation into the polymer structure when supplemented by proper structural precursors was tested by adding propanol, pentanol, propionate, valerate or levulinate to the PHA production medium at a conc. of 2 g/L after 24 h of cultivation with mannitol.

To assess the *i-phaZ* deletion mutants ability to access intracellular PHA as carbon and energy source, it was subjected to PHA-accumulation conditions as described earlier. Therefore, prepared PHB-filled cells were washed with the PHA production medium without the primary carbon source and then introduced into the same medium at 10% v/v. Wild-type A1169 was used as a control strain. Cell growth was monitored during the 96 h incubation period by colony forming unit count on R3A agar at 15 °C.

### Biomass analysis

Ten mL of bacterial suspension was collected after the incubation period on PHA production medium, centrifuged at 6000×*g* for 5 min, washed with distilled water and dried at 80 °C until constant mass was achieved. PHA composition and content of the dried biomass were determined by gas chromatography with a flame ionization detector (GC-FID) as described previously (Obruca et al. 2013).

### Data analysis

Illumina reads were deposited in the NCBI Sequence Read Archive (SRA) as BioProject PRJNA991094. All results were compiled using Excel 2016 (MS Office) for Windows. Data visualization and statistical analysis have been performed using the R software (R v.4.2.3) and the following packages: ggplot2, ggpubr, (R Core Team 2002). Phylogenetic trees were made using the Mega-174 X software.

## Results and discussion

### Substrate preference of the strain *Acidovorax* sp. A1169

**Fig. 1:**
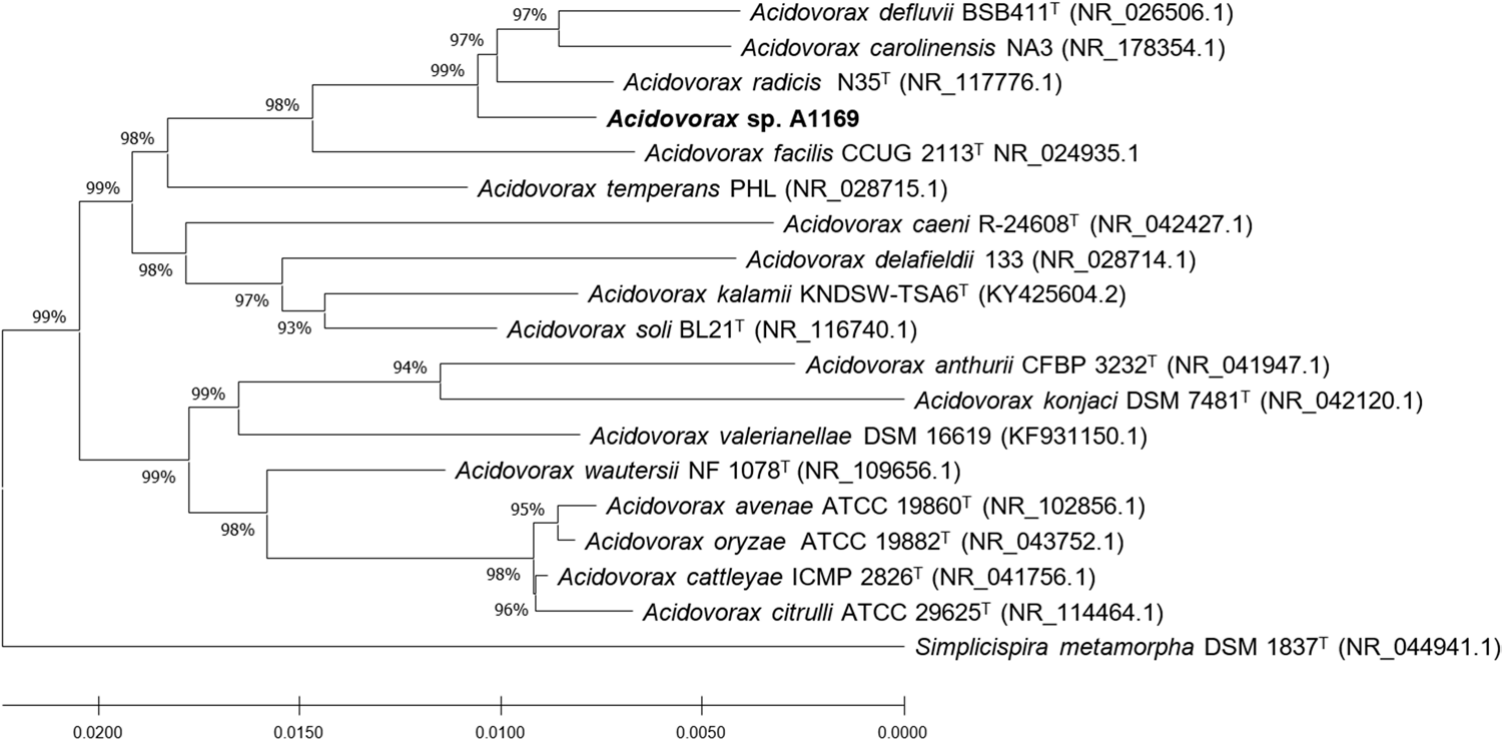
Neighbor-joining phylogenetic tree based on 16S rRNA gene sequences showing the position of strain A1169 and related species in the genus *Acidovorax* (family Comamonadaceae). Numbers at nodes are bootstrap percentages based on the neighbor-joining algorithm. Simplicispira metamorpha DSM 1837T was used as an outgroup. Sequences were retrieved from the NCBI database. Bar shows substitutions per nucleotide position

**Fig. 2:**
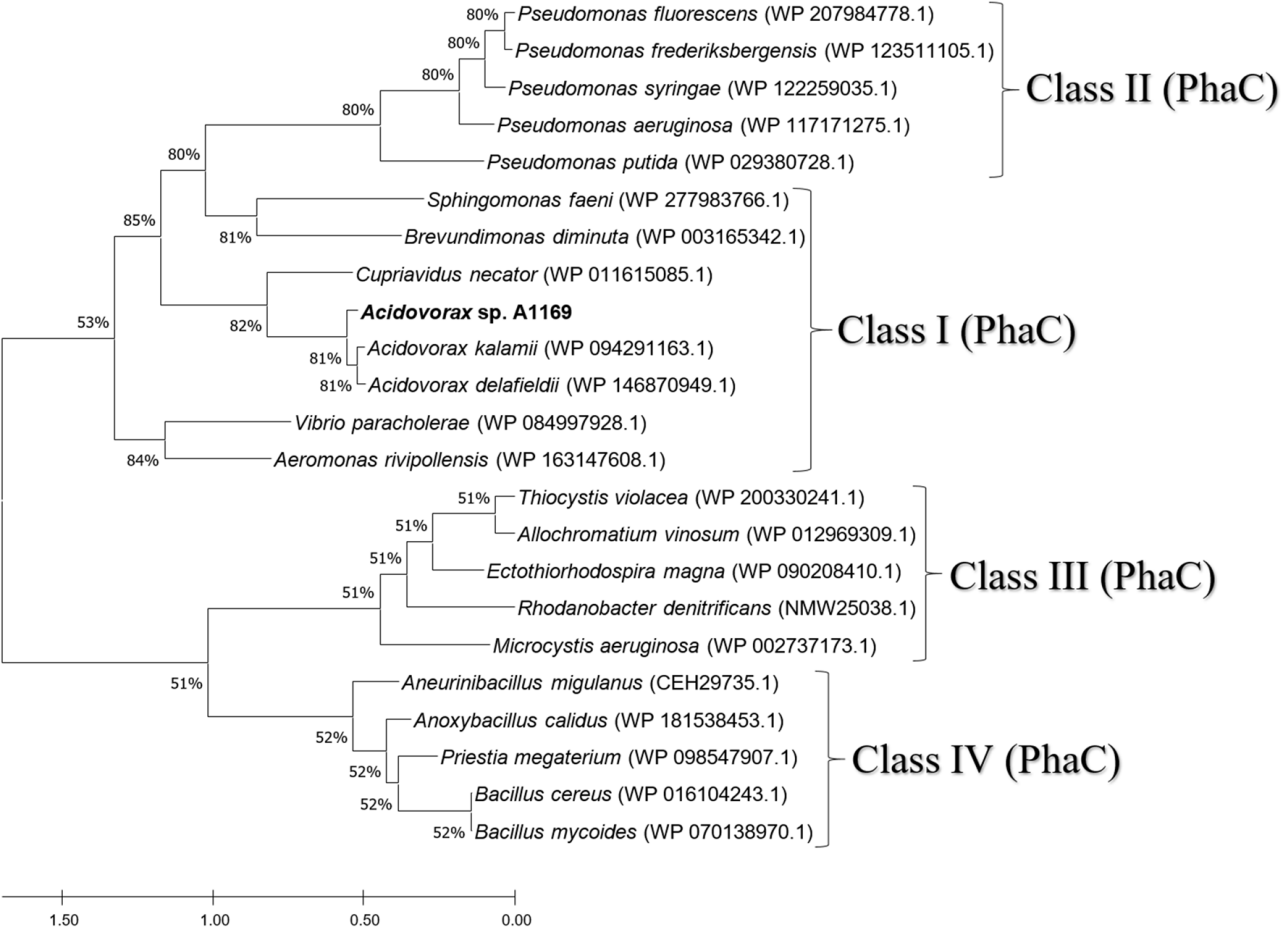
Neighbor-joining phylogenetic tree based on *phaC* (polyhydroxyalkanoic acid synthase) amino acid sequences showing the position of strains A1169 *phaC* sequence among other *phaC* sequences belonging to four synthase classes. Sequences were retrieved from the NCBI database. Bar shows substitutions per amino acid position. Numbers at nodes are bootstrap percentages based on the neighbor-joining algorithm

**Fig. 3:**
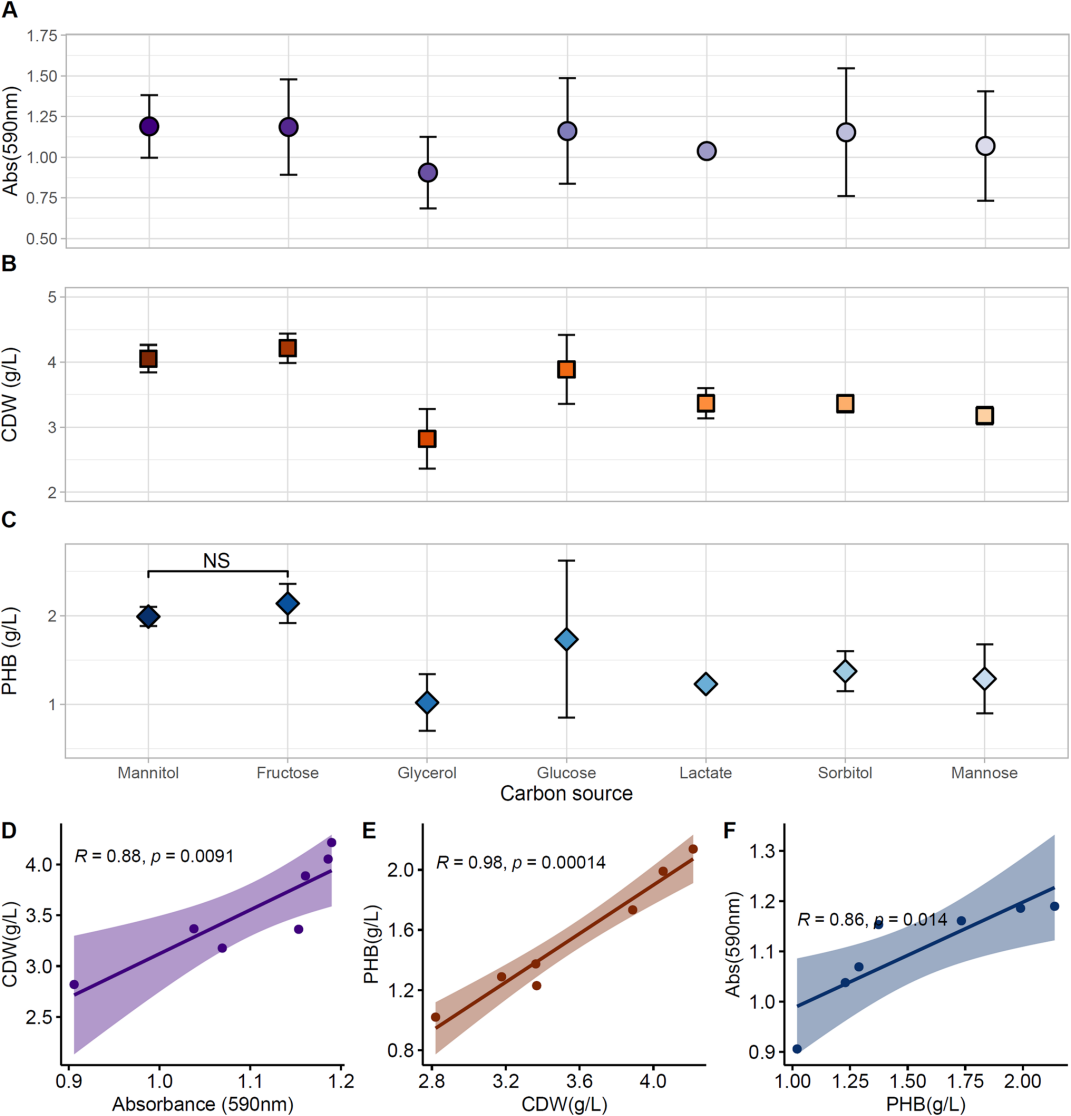
Carbon source utilization by *Acidovorax* sp. A1169 based on **a** the GENIII Microplate respiratory assay; **b** cellular dry mass (CDW) concentration; **c** PHB concentration. Correlation between variables: **d** GENIII microplate and cell biomass; **e** cellular dry mass (CDW) and PHB; **f** PHB and GENIII microplate

The genus *Acidovorax* (family *Comamonadaceae*, class *Betaproteobacteria*) currently comprises 20 validly published species (Du et al. 2023). 16S rRNA gene sequence similarity with sequences of the aforementioned species deposited in the NCBI blastn database revealed that strain A1169 shared a 99.4% and 99.02% similarity with *A.* *radicis* and *A.* *defluvii*, respectively (Fig. 1), not meeting the conditions for a new species (Kim et al. 2014). However, its placement on a separate branch of the phylogenetic tree and also its Arctic glacier origin suggest a new quality within the genus (Du et al. 2023). *Acidovorax* sp. A1169 genome annotation revealed the presence of one gene coding for a PHA synthase. The placement of its deduced amino acid sequence suggests it belongs to class I PHA synthases (Fig. 2) capable of synthesizing scl-PHAs as was further confirmed by GC analysis of *Acidovorax* sp. A1169 biomass when various compounds were supplied as substrates (Fig. 3c).

Generally, isolates of the genus *Acidovorax* were obtained from different sources, both environmental (soil, rhizosphere, water, activated sludge) and clinical, hinting that phenotypic and genotypic features vary considerably between species/strains (Willems 2014). Therefore, the employment of phenotypic microarrays like the Biolog GENIII microplate can be justified as it provides an effective and reliable tool for screening the bacterium with respect to utilization of various carbon compounds and other cultivation conditions for environmental bacteria (Woźniak et al. 2019). However, the main purpose of this system is bacterial identification based on the carbon source utilization pattern and other metabolic features provided in the 96-well plates by the manufacturer (Sandle et al. 2013). On rare occasions, it has been used to ascertain a set of carbon compounds that support growth in microbiological media (DeAngelis et al. 2011). Seven compounds were revealed in the GENIII microplate assay as supporting high respiratory rates in *Acidovorax* sp. A1169 (Fig. 3a). Highest values were noted for mannitol (*A*_590_ = 1.19 ± 0.19) and fructose (*A*_590_ = 1.19 ± 0.29), while the lowest within this group for glycerol (*A*_590_ = 0.91 ± 0.22). Biomass concentration analysis produced similar results when carbon sources were supplied into the PHA production medium at a concentration of 10 g/L (Fig. 3b). When A1169 was cultivated on mannitol and fructose the following biomass concentrations was achieved (g/L): 4.05 ± 0.21 and 4.22 ± 0.23, respectively, while on glycerol biomass concentration reached 2.82 ± 0.46. The highest PHB concentration was achieved on fructose (2.14 ± 0.22 g/L) followed by mannitol (1.99 ± 0.11 g/L) corresponding to 50.7% and 49.1% of PHB in cell dry mass, respectively (Fig. 3c). There was a positive, significant correlation between values of the GENIII assay, dry biomass concentration and PHB content at *p* ≥ 0.05 (Fig. 3d–f). Correlations between respiration rates and PHB concentration suggest Biolog GENIII microplates as a suitable guiding tool towards optimization of bacterial biomass and PHA accumulation.

Hence, the highest PHB yield was achieved for the A1169 strain on fructose and mannitol. Fructose has proven to be the best carbon source for the flag PHB producer *C*. *necator* as well as the halophile *Halomonas* sp. YLGW01 (Nygaard et al. 2021; Park et al. 2020). Besides the examined here *Acidovorax* sp., other members of the family *Comamonadaceae* were also able to turn fructose into PHB, however, other sources like glucose or xylose yielded higher concentrations of the biopolymer (Yamaguchi et al. 2019; Kourilova et al. 2020, 2021a). Nevertheless, even fructose can be considered as sustainable and renewable carbon substrate for PHA production when derived from properly chosen resources. For instance, Corrado et al*.* recently described PHA synthesis form hydrolysates of waste inulin rich in fructose content (Corrado et al. 2021). Mannitol was rarely considered as a substrate for PHB biosynthesis, although some mannitol-rich agro-industrial wastes such as celery waste (*Apium* *graveolens*) and ensiled grass press juice were recognized as potent substrates for PHA production employing *Cobetia* *amphilecti* and *Burkholderia* *sacchari* as producers (Cerrone et al. 2015; Gnaim et al. 2022).

### Effect of temperature and cultivation time on growth and PHA synthesis in Acidovorax sp. A1169

**Fig. 4:**
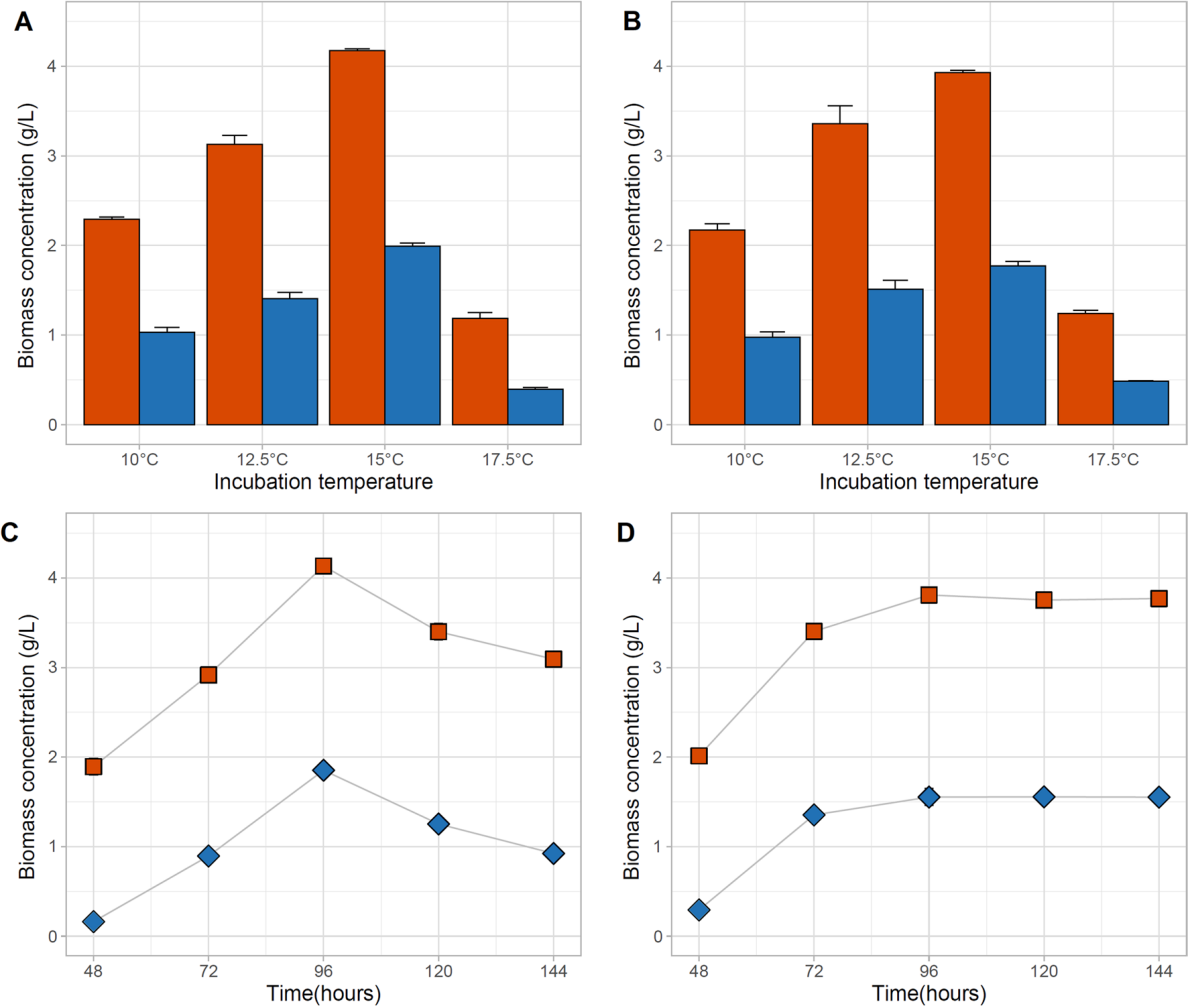
Effects of incubation temperature on biomass concentration using **a** fructose as carbon source; **b** mannitol as carbon source. Biomass concentration changes during cultivation when fructose (**c**) or mannitol (**d**) was the carbon source. Orange—cellular dry mass (CDW); blue—PHB

Incubation temperature had a considerable effect on the biomass and also on the PHB concentration in *Acidovorax* sp. A1169 culture (Fig. 4a, b). The highest PHA titers were achieved at 15 °C on fructose [4.17 ± 0.02 g/L CDW (cellular dry weight) and 1.99 ± 0.03 g/L PHB] and mannitol (3.93 ± 0.02 g/L CDW and 1.77 ± 0.05 g/L PHB) as substrate. At 17.5 °C biomass and PHB concentrations were the lowest, on fructose: 1.19 ± 0.06 g/L CDW and 0.39 ± 0.02 g/L PHB), while for mannitol: 1.24 ± 0.03 g/L CDW and 0.49 ± 0.002 g/L PHB. Such a low optimal temperature of PHB biosynthesis has not been observed before, even when polar-region or high-altitude strains were tested (Kumar et al. 2020; Pacheco et al. 2019; Choi et al. 2021). Arctic and Antarctic *Pseudomonas* strains for example displayed the highest PHB production rates at 30 °C, a temperature that is outside the range of what is considered psychrophilic (Pacheco et al. 2019; Choi et al. 2021; Madigan et al. 2019). In the case of *Acidovorax* sp. A1169, the increase in incubation temperature by 2.5 °C caused not only a 77% drop in PHB yield, but also an approx. 66% decreases in the PHB-free biomass concentration, hinting that not only the enzymatic machinery behind PHB biosynthesis was affected but also essential cellular metabolism. The time frame of PHA synthesis is one of many factors that affect the efficiency of biopolymer production (Koller et al. 2010, 2017). The impact of incubation time on biomass production of strain A1169 varied between carbon sources (Fig. 4c, d). The highest values of biomass and PHB concentrations were achieved for both, fructose and mannitol after 96 h, yet for fructose these values dropped afterward, while for mannitol they remained relatively stable. *C*. *necator* achieved maximal PHB titers in flasks within 72 h at 30 °C while a variety of thermophiles display a similar PHB biosynthesis period at 50–55 °C (Obruca et al. 2013; Aramvash et al. 2015; Nygaard et al. 2021; Kourilova et al. 2020, 2021a; b). Considering that the specific growth rate of meso- and thermophiles is approx. 2.5 × higher than psychrophiles, the additional 24 h required by strain A1169 to achieve maximal PHB concentration can be considered efficient (Mohr and Krawiec 1980) and the bacterium *Acidivorax* so. A1169 can be considered as promising psychrophilic bacterium for PHA production aligning to the concept of Next-Generation Industrial Biotechnology (Chen and Jiang 2018).

### Identification of suitable nitrogen source and carbon source concentration

**Fig. 5:**
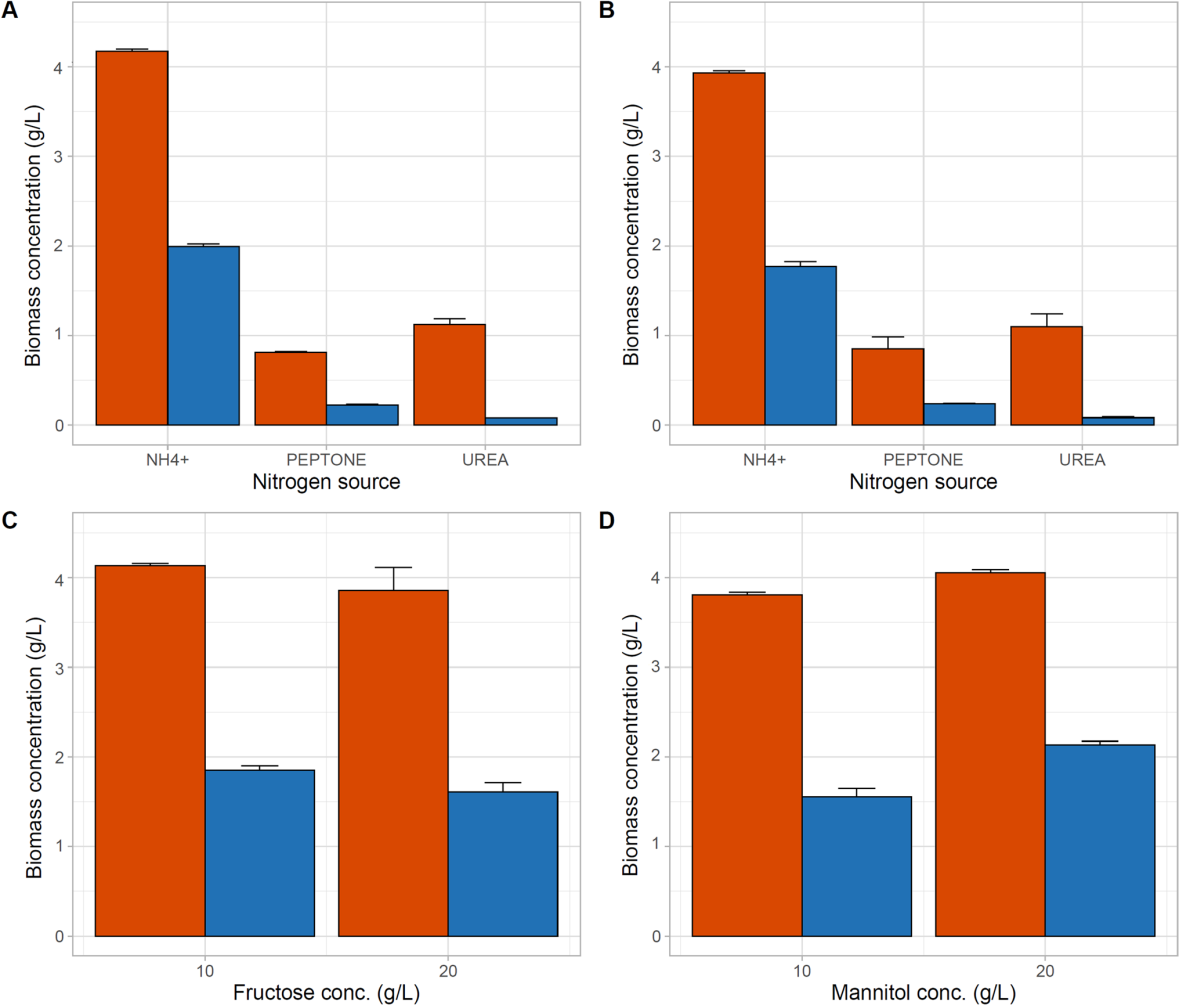
Effects of different nitrogen sources on biomass concentration using **a** fructose as carbon source; **b** mannitol as carbon source. Effects of carbon source concentration on the concentration of produced biomass (**c**, **d**). Orange—cellular dry mass (CDW); blue—PHB

Replacing the ammonium chloride with peptone or urea greatly lowered the biomass and subsequently the PHB concentration in *Acidovorax* sp. A1169 culture (Fig. 5a, b). Peptone stimulated higher PHB synthesis with lower cell dry weight (fructose: 0.82 ± 0.01 g CDW/L and 0.23 ± 0.003 g PHB/L; mannitol: 0.85 ± 0.13 g CDW/L and 0.24 ± 0.002 g PHB/L), whereas with urea the opposite was the case (fructose: 1.16 ± 0.01 g CDW/L and 0.1 ± 0.0002 g PHB/L; mannitol: 1.1 ± 0.14 g CDW/L and 0.01 ± 0.001 g PHB/L). As with the carbon source, nitrogen source effect seems to be bacteria species specific, with peptone and/or urea being preferable over ammonia salts by *Vibrio* *proteolyticus*, *Pseudomonas* *aeruginosa* and *Erythrobacter* *aquimaris*, while *Rhizobium* *etli* and *Pseudomonas* *stutzeri* preferred ammonia salts over peptone or even yeast extract (Hong et al. 2019; Tripathi et al. 2012; Mostafa et al. 2020; Belal 2013).

The concentration of the carbon source—fructose or mannitol had an interesting effect on biomass concentrations (Fig. 5c, d). In the case of fructose, biomass and PHB yield decreased with higher substrate concentration, whereas with mannitol it raised. However, the highest amounts of PHB per gram of supplied substrate were achieved for the conc. of 10 g/L: fructose—0.19 g PHB per g substrate, mannitol—0.16 g PHB per g substrate, while at 20 g/L: fructose—0.08 g PHB per g substrate, mannitol—0.1 g PHB per g substrate. This phenomenon of PHB biosynthesis suppression at relatively high substrate concentration has been observed before, albeit the optimal carbon source concentration being 20 g/L or higher, with *C*. *necator* managing 40 g/L without adverse effects (Wendy et al. 2022; Hong et al. 2019; Sriyapai et al. 2022; Nygaard et al. 2021). Growth and PHB production decrease in higher substrate concentration can be explained by osmotic and/or nutrient shock (Azevedo et al. 2012). The glacial origin of the strain may suggest both as glacier-hosted habitats are often poor in nutrients and solutes (Grzesiak et al. 2015).

### Characterization of i-phaZ deletion mutant

**Fig. 6:**
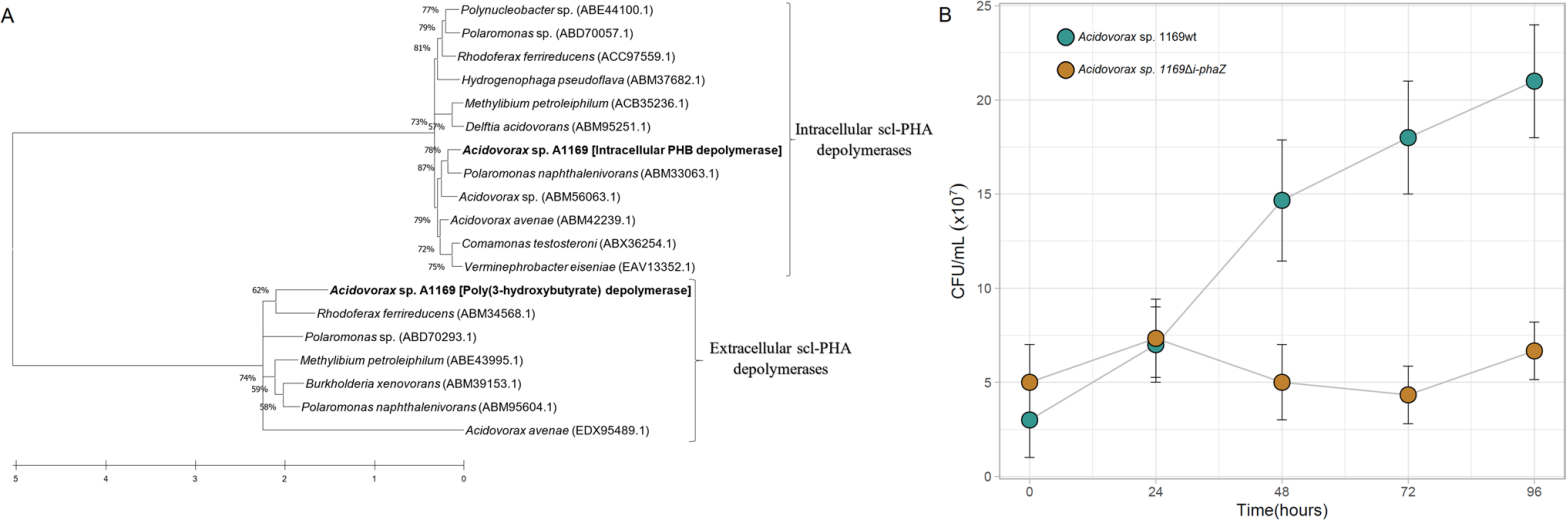
**a** Neighbor-joining phylogenetic tree based on PHASCL depolymerases amino acid sequences showing the positions of two PHASCL depolymerases found within the genome of strain A1169. Sequences were retrieved from the NCBI database and their identity as internal or external depolymerases was confirmed with The PHA Depolymerase Engineering Database (Knoll et al. 2009). Bar shows substitutions per amino acid position. Numbers at nodes are bootstrap percentages based on the neighbor-joining algorithm. **b** Cell abundance dynamics of *Acidovorax* sp. A1169 wild type strain and its i-phaZ gene knockout mutant in carbon source-lacking mineral medium. The cells were introduced into the medium after a 96 h incubation in PHB-accumulation inducing conditions

**Fig. 7:**
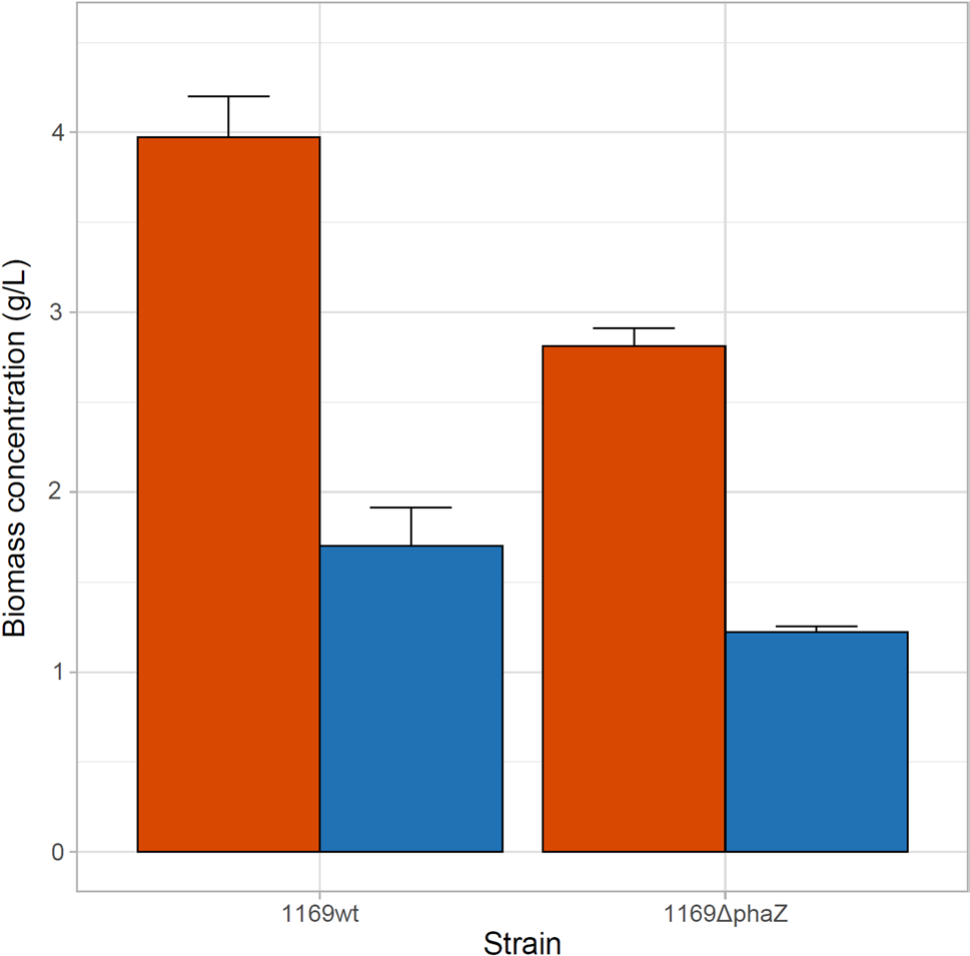
Comparison of biomass concentration between *Acidovorax* sp. A1169 and its i-phaZ gene knock-out mutant cultivated in the same conditions. Orange—cellular dry mass (CDW); blue—PHB

Two PHB-depolymerase genes were recognized in the genome of *Acidovorax* sp. A1169. Analysis based on a phylogenetic neighbor-joining tree amended with amino acid sequences of confirmed intracellular and extracellular scl-PHA depolymerases revealed that one of the recognized genes coding for an intracellular depolymerase (Fig. 6a) (Knoll et al. 2009). As the inactivation of this enzyme is one of the ways to enhance PHA yields (Wang et al. 2023), an *Acidovorax* sp. A1169 deletion mutant in the *i-phaZ* gene was constructed. The PHB-filled mutant failed to increase in numbers while being incubated in a mineral medium lacking a carbon source contrary to the PHB-filled wild-type strain confirming its inability to use intracellular PHB as energy and carbon supply (Fig. 6b). Biomass and PHB accumulation was considerably lower in the *i-phaZ* gene deletion mutant than in the wild A1169 strain when cultured in the same PHB-accumulation promoting conditions. While 1.71 ± 0.21 g PHB/L was achieved for the wild-type strain only 1.22 ± 0.03 g PHB/L was accumulated by the mutant strain (Fig. 7), with both displaying 43% PHB contribution to the CDW. There were several instances of a successful enhancement of the PHA yield by employing PHA-depolymerase negative mutants when compared to the wild-type strains although some reported a decreased growth strength of the former. The reduced anabolic performance of the *Acidovorax* sp. A1169Δ*i-phaZ* mutant might indicate a strong degree of interaction between PHB metabolism and its other metabolic pathways. Various phenotypic effects were observed after *i-phaZ* deactivation in other bacterial species, most notably, the sensitivity to different stressors being enhanced (Kadouri et al. 2003; Handrick et al. 2000). Intracellular PHA depolymerases are known to interact with other PHA granule-associated proteins, like phasins which dictate the size and numbers of PHA granules but can also regulate the amount of PHA synthase enzyme in the cell (Mezzina and Pettinari 2016). Furthermore, the interplay between PHA synthesizing and degrading enzymes has a profound influence on the energy potential of the bacterial cell by shifting the available acetyl-CoA/free CoA and NAD(P)H/NAD(P) ratios (Kessler and Witholt 2001). Therefore, it seems that complete PHA metabolism is crucial for *Acidovorax* *sp.* A1169 with respect to its PHA synthesis capacity and deletion of the gene encoding for *i-phaZ* harms the overall robustness of the bacterium.

### Synthesis of PHA copolymers by *Acidovorax* sp. A1169

**Fig. 8:**
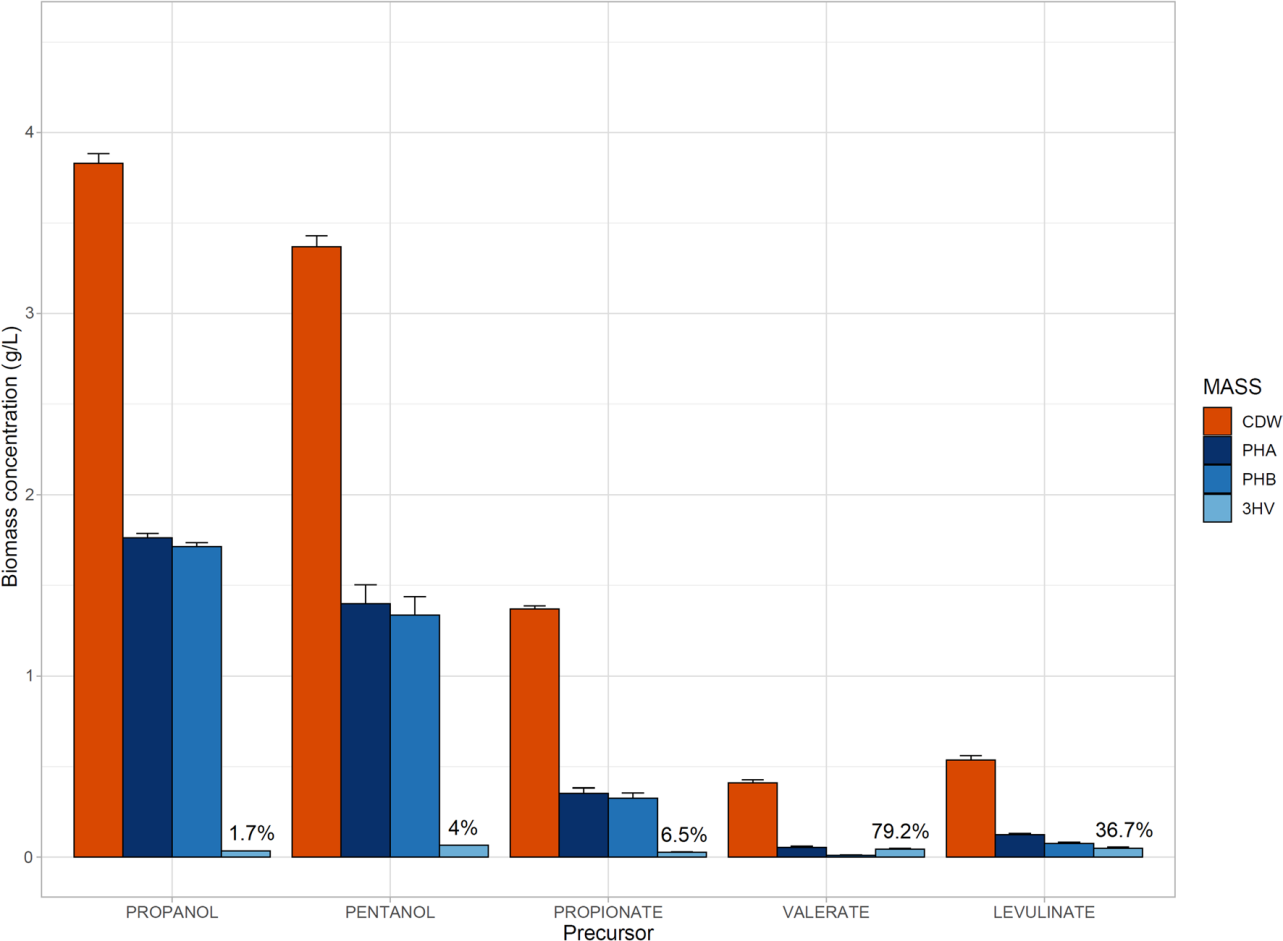
Effects of different 3HV precursor addition on the quality and quantity of PHA produced by *Acidovorax* sp. A1169

The addition of different 3HV precursors had a varying effect on the whole biomass concentration and PHB yield (Fig. 8). The highest copolymer titer was achieved on pentanol with approx. 4 mol% of 3HV at 1.40 g PHA/L, albeit the presence of the alcohol decreased the growth of the bacterial biomass when compared to the values obtained for propanol. Propionate, valerate and levulinate severely hampered the growth of A1169 strain, which was observed before in other bacterial species (Kourilova et al. 2021a, b). However, despite the relatively low biomass, PHA had a substantial 3HV molar contribution of 79.2% and 36.7% on valerate and levulinate as precursors, respectively. Based on the findings of Kourilova and colleagues, such results indicate, that the growth of the bacterium in the presence of the 3HV precursor can be further optimized to achieve greater yields of the co-polymer by carefully adjusting the precursor amount and the timing of its introduction into the medium (Kourilova et al. 2020).

## Conclusions

Our findings demonstrate the remarkable potential of *Acidovorax* sp. A1169, which can be considered as a potent PHA producer under low temperatures and somewhat oligotrophic conditions, aligning perfectly with the Next-Generation Industrial Biotechnology concept. To the best of our knowledge, this study represents the first comprehensive description that offers insights into a novel truly low-temperature PHA biosynthesis process. It not only provides guidelines for exploring polar-region bacteria as a valuable resource for PHA production but also highlights the methodology for the genetic engineering of psychrophiles. Although this research screened the most essential cultivation conditions for PHA production employing *Acidovorax* sp. A1169, further investigations are necessary to accurately assess its industrial-scale synthesis potential. The utilization of the bacterium for PHA production holds promise not only in terms of energy efficiency but also in the valorization of low-concentration effluents associated with carbohydrate processing (e.g., high fructose corn syrup production, inulin processing), which would otherwise be discarded.

## Data Availability

The data sets generated and/or analysed in the current study are available in the NCBI repository, BioProject number PRJNA991094. Submission information can be found at: https://www.ncbi.nlm.nih.gov/sra.

## Code availability

Codes used in the current study are available from the corresponding author upon reasonable request.

## References

[CR1] Andrews S (2010) FastQC: a quality control tool for high throughput sequence data. https://www.bioinformatics.babraham.ac.uk/projects/fastqc

[CR2] Aramvash A, Akbari Shahabi Z, Dashti Aghjeh S, Ghafari MD (2015). Statistical physical and nutrient optimization of bioplastic polyhydroxybutyrate production by *Cupriavidus*
*necator*. Int J Environ Sci Technol.

[CR3] Azevedo NF, Bragança SM, Simões LC, Cerqueira L, Almeida C, Keevil CW, Vieira MJ (2012). Proposal for a method to estimate nutrient shock effects in bacteria. BMC Res Notes.

[CR4] Belal EB (2013). Production of poly-β-hydroxybutyric acid (PHB) by *Rhizobium*
*elti* and *Pseudomonas*
*stutzeri*. Curr Res J Biol.

[CR5] Cerrone F, Davis R, Kenny ST, Woods T, O’Donovan A, Gupta VK (2015). Use of a mannitol rich ensiled grass press juice (EGPJ) as a sole carbon source for polyhydroxyalkanoates (PHAs) production through high cell density cultivation. Bioresour Technol.

[CR6] Chen GQ, Jiang XR (2018). Next generation industrial biotechnology based on extremophilic bacteria. Curr Opin Biotechnol.

[CR500] Chen S, Zhou Y, Chen Y, Gu J (2018). fastp: an ultra-fast all-in-one FASTQ preprocessor. Bioinformatics.

[CR7] Choi TR, Park YL, Song HS, Lee SM, Park SL, Lee HS (2021). Fructose-based production of short-chain-length and medium-chain-length polyhydroxyalkanoate copolymer by arctic *Pseudomonas* sp. B14–6. Polymers.

[CR8] Ciesielski S, Górniak D, Możejko J, Świątecki A, Grzesiak J, Zdanowski M (2014). The diversity of bacteria isolated from Antarctic freshwater reservoirs possessing the ability to produce polyhydroxyalkanoates. Curr Microbiol.

[CR9] Corrado I, Petrillo C, Isticato R, Casillo A, Corsaro MM, Sannia G, Pezzella C (2021). The power of two: an artificial microbial consortium for the conversion of inulin into polyhydroxyalkanoates. Int J Biol Macromol.

[CR10] DeAngelis KM, D’Haeseleer P, Chivian D, Fortney JL, Khudyakov J, Simmons B (2011). Complete genome sequence of “*Enterobacter*
*lignolyticus*” SCF1. Stand Genom Sci.

[CR11] Du J, Liu Y, Zhu H (2023). Genome-based analyses of the genus *Acidovorax*: proposal of the two novel genera *Paracidovorax* gen. nov., *Paenacidovorax* gen. nov. and the reclassification of *Acidovorax*
*antarcticus* as *Comamonas*
*antarctica* comb. nov. and emended description of the genus *Acidovorax*. Arch Microbiol.

[CR12] El-Sayed AK, Hothersall J, Thomas CM (2001). Quorum-sensing-dependent regulation of biosynthesis of the polyketide antibiotic mupirocin in *Pseudomonas*
*fluorescens* NCIMB 10586. Microbiology.

[CR13] Gawor J, Grzesiak J, Sasin-Kurowska J, Borsuk P, Gromadka R, Górniak D (2016). Evidence of adaptation, niche separation and microevolution within the genus *Polaromonas* on Arctic and Antarctic glacial surfaces. Extremophiles.

[CR14] Georlette D, Blaise V, Collins T, D'Amico S, Gratia E, Hoyoux A (2004). Some like it cold: biocatalysis at low temperatures. FEMS Microbiol Rev.

[CR15] Gnaim R, Unis R, Gnayem N, Das J, Gozin M, Golberg A (2022). Turning mannitol-rich agricultural waste to poly(3-hydroxybutyrate) with *Cobetia*
*amphilecti* fermentation and recovery with methyl levulinate as a green solvent. Bioresour Technol.

[CR16] Goh YS, Tan IKP (2012). Polyhydroxyalkanoate production by Antarctic soil bacteria isolated from Casey Station and Signy Island. Microbiol Res.

[CR17] Grzesiak J, Górniak D, Świątecki A, Aleksandrzak-Piekarczyk T, Szatraj K, Zdanowski MK (2015). Microbial community development on the surface of Hans and Werenskiold Glaciers (Svalbard, Arctic): a comparison. Extremophiles.

[CR18] Handrick R, Reinhardt S, Jendrossek D (2000). Mobilization of poly (3-hydroxybutyrate) in *Ralstonia*
*eutropha*. J Bacteriol.

[CR19] Hong JW, Song HS, Moon YM, Hong YG, Bhatia SK, Jung HR (2019). Polyhydroxybutyrate production in halophilic marine bacteria *Vibrio*
*proteolyticus* isolated from the Korean peninsula. Bioprocess Biosyst Eng.

[CR20] Inoue H, Nojima H, Okayama H (1990). High efficiency transformation of *Escherichia*
*coli* with plasmids. Gene.

[CR21] Kadouri D, Jurkevitch E, Okon Y (2003). Poly β-hydroxybutyrate depolymerase (PhaZ) in *Azospirillum*
*brasilense* and characterization of a phaZ mutant. Arch Microbiol.

[CR22] Kessler B, Witholt B (2001). Factors involved in the regulatory network of polyhydroxyalkanoate metabolism. J Biotechnol.

[CR23] Kim M, Oh HS, Park SC, Chun J (2014). Towards a taxonomic coherence between average nucleotide identity and 16S rRNA gene sequence similarity for species demarcation of prokaryotes. Int J Syst Evol Microbiol.

[CR24] Knoll M, Hamm TM, Wagner F, Martinez V, Pleiss J (2009). The PHA depolymerase engineering database: a systematic analysis tool for the diverse family of polyhydroxyalkanoate (PHA) depolymerases. BMC Bioinform.

[CR25] Koller M (2017). Production of polyhydroxyalkanoate (PHA) biopolyesters by extremophiles. MOJ Polym Sci.

[CR26] Koller M (2018). Chemical and biochemical engineering approaches in manufacturing polyhydroxyalkanoate (PHA) biopolyesters of tailored structure with focus on the diversity of building blocks. Chem Biochem Eng Q.

[CR27] Koller M, Salerno A, Dias M, Reiterer A, Braunegg G (2010). Modern biotechnological polymer synthesis: a review. Food Technol Biotechnol.

[CR28] Koller M, Maršálek L, de Sousa Dias MM, Braunegg G (2017). Producing microbial polyhydroxyalkanoate (PHA) biopolyesters in a sustainable manner. Nat Biotechnol.

[CR29] Kourilova X, Pernicova I, Sedlar K, Musilova J, Sedlacek P, Kalina M (2020). Production of polyhydroxyalkanoates (PHA) by a thermophilic strain of *Schlegelella*
*thermodepolymerans* from xylose rich substrates. Bioresour Technol.

[CR30] Kourilova X, Pernicova I, Vidlakova M, Krejcirik R, Mrazova K, Hrubanova K (2021). Biotechnological conversion of grape pomace to poly(3-hydroxybutyrate) by moderately thermophilic bacterium *Tepidimonas*
*taiwanensis*. Bioengineering.

[CR31] Kouřilová X, Schwarzerová J, Pernicová I, Sedlář K, Mrázová K, Krzyžánek V (2021). The first insight into polyhydroxyalkanoates accumulation in multi-extremophilic *Rubrobacter*
*xylanophilus* and *Rubrobacter*
*spartanus*. Microorganisms.

[CR32] Kumar V, Thakur V, Kumar V, Kumar R, Singh D (2020). Genomic insights revealed physiological diversity and industrial potential for *Glaciimonas* sp. PCH181 isolated from Satrundi glacier in Pangi-Chamba Himalaya. Genomics.

[CR33] Kumar V, Thakur V, Ambika, Kumar S, Singh D (2018) Bioplastic reservoir of diverse bacterial communities revealed along altitude gradient of Pangi-Chamba trans-Himalayan region. FEMS Microbiol Lett 365(14):fny144 10.1093/femsle/fny144 29912320

[CR34] Madigan MT, Bender KS, Buckley DH, Sattley WM, Stahl DA (2019). Brock biology of microorganisms.

[CR35] Margesin R, Schinner F, Marx JC, Gerday C (2008). Psychrophiles: from biodiversity to biotechnology.

[CR36] Mezzina MP, Pettinari MJ (2016). Phasins, multifaceted polyhydroxyalkanoate granule-associated proteins. Appl Environ Microbiol.

[CR37] Mohr PW, Krawiec S (1980). Temperature characteristics and Arrhenius plots for nominal psychrophiles, mesophiles and thermophiles. Microbiology.

[CR38] Moradali MF, Rehm BH (2020). Bacterial biopolymers: from pathogenesis to advanced materials. Nat Rev Microbiol.

[CR39] Mostafa YS, Alrumman SA, Otaif KA, Alamri SA, Mostafa MS, Sahlabji T (2020). Production and characterization of bioplastic by polyhydroxybutyrate accumulating *Erythrobacter*
*aquimaris* isolated from mangrove rhizosphere. Molecules.

[CR40] Możejko-Ciesielska J, Kiewisz R (2016). Bacterial polyhydroxyalkanoates: still fabulous?. Microbiol Res.

[CR41] Müller-Santos M, Koskimäki JJ, Alves LPS, de Souza EM, Jendrossek D, Pirttilä AM (2021). The protective role of PHB and its degradation products against stress situations in bacteria. FEMS Microbiol Rev.

[CR42] Nowroth V, Marquart L, Jendrossek D (2016). Low temperature-induced viable but not culturable state of *Ralstonia*
*eutropha* and its relationship to accumulated polyhydroxybutyrate. Microbiol Lett.

[CR43] Nygaard D, Yashchuk O, Noseda DG, Araoz B, Hermida ÉB (2021). Improved fermentation strategies in a bioreactor for enhancing poly(3-hydroxybutyrate)(PHB) production by wild type *Cupriavidus*
*necator* from fructose. Heliyon.

[CR44] Obruca S, Snajdar O, Svoboda Z, Marova I (2013). Application of random mutagenesis to enhance the production of polyhydroxyalkanoates by *Cupriavidus*
*necator* H16 on waste frying oil. World J Microbiol Biotechnol.

[CR45] Obruca S, Sedlacek P, Krzyzanek V, Mravec F, Hrubanova K, Samek O (2016). Accumulation of poly (3-hydroxybutyrate) helps bacterial cells to survive freezing. PLoS ONE.

[CR46] Pacheco N, Orellana-Saez M, Pepczynska M, Enrione J, Bassas-Galia M, Borrero-de Acuña JM (2019). Exploiting the natural poly (3-hydroxyalkanoates) production capacity of Antarctic *Pseudomonas* strains: from unique phenotypes to novel biopolymers. J Ind Microbiol Biotechnol.

[CR47] Park YL, Bhatia SK, Gurav R, Choi TR, Kim HJ, Song HS (2020). Fructose based hyper production of poly-3-hydroxybutyrate from *Halomonas* sp. YLGW01 and impact of carbon sources on bacteria morphologies. Int J Biol Macromol.

[CR48] Reddy CSK, Ghai R, Kalia V (2003). Polyhydroxyalkanoates: an overview. Biores Technol.

[CR49] Rehakova V, Pernicova I, Kourilova X, Sedlacek P, Musilova J, Sedlar K (2023). Biosynthesis of versatile PHA copolymers by thermophilic members of the genus *Aneurinibacillus*. Int J Biol Macromol.

[CR50] Rogala MM, Gawor J, Gromadka R, Kowalczyk M, Grzesiak J (2020). Biodiversity and habitats of polar region polyhydroxyalkanoic acid-producing bacteria: bioprospection by popular screening methods. Genes.

[CR51] Sandle T, Skinner K, Sandle J, Gebala B, Kothandaraman P (2013). Evaluation of the GEN III OmniLog® ID System microbial identification system for the profiling of cleanroom bacteria. Eur J Parenter Pharm Sci.

[CR52] Smorawinska M, Szuplewska M, Zaleski P, Wawrzyniak P, Maj A, Plucienniczak A, Bartosik D (2012). Mobilizable narrow host range plasmids as natural suicide vectors enabling horizontal gene transfer among distantly related bacterial species. FEMS Microbiol Lett.

[CR53] Sriyapai T, Chuarung T, Kimbara K, Samosorn S, Sriyapai P (2022). Production and optimization of polyhydroxyalkanoates (PHAs) from *Paraburkholderia* sp. PFN 29 under submerged fermentation. Electron J Biotechnol.

[CR54] Tan D, Wang Y, Tong Y, Chen GQ (2021). Grand challenges for industrializing polyhydroxyalkanoates (PHAs). Trends Biotechnol.

[CR55] Tripathi AD, Yadav A, Jha A, Srivastava SK (2012). Utilizing of sugar refinery waste (cane molasses) for production of bio-plastic under submerged fermentation process. J Polym Environ.

[CR56] Tripathi AD, Srivastava SK, Singh RP (2013). Statistical optimization of physical process variables for bio-plastic (PHB) production by *Alcaligenes* sp. Biomass Bioenergy.

[CR57] Wang Z, Zheng Y, Ji M, Zhang X, Wang H, Chen Y (2022). Hyperproduction of PHA copolymers containing high fractions of 4-hydroxybutyrate (4HB) by outer membrane-defected *Halomonas*
*bluephagenesis* grown in bioreactors. Microb Biotechnol.

[CR58] Wang J, Liu S, Huang J, Cui R, Xu Y, Song Z (2023). Genetic engineering strategies for sustainable polyhydroxyalkanoate (PHA) production from carbon-rich wastes. Environ Technol Innov.

[CR59] Weimer A, Kohlstedt M, Volke DC, Nikel PI, Wittmann C (2020). Industrial biotechnology of *Pseudomonas*
*putida*: advances and prospects. Appl Microbiol Biotechnol.

[CR60] Wendy YD, Fauziah MN, Baidurah YS, Tong WY, Lee CK (2022). Production and characterization of polyhydroxybutyrate (PHB) by *Burkholderia*
*cepacia* BPT1213 using waste glycerol as carbon source. Biocatal Agric Biotechnol.

[CR61] Willems A, Rosenberg E, DeLong EF, Lory S, Stackebrandt E, Thompson F (2014). The family Comamonadaceae. The prokaryotes: Alphaproteobacteria and Betaproteobacteria.

[CR62] Wilson K (2001). Preparation of genomic DNA from bacteria. Curr Protoc Mol Biol.

[CR63] Wolfenden R, Yuan Y (2008). Rates of spontaneous cleavage of glucose, fructose, sucrose, and trehalose in water and the catalytic proficiencies of invertase and trehalase. J Am Chem Soc.

[CR64] Woźniak M, Gałązka A, Tyśkiewicz R, Jaroszuk-Ściseł J (2019). Endophytic bacteria potentially promote plant growth by synthesizing different metabolites and their phenotypic/physiological profiles in the Biolog GEN III MicroPlateTM Test. Int J Mol Sci.

[CR65] Yamaguchi T, Narsico J, Kobayashi T, Inoue A, Ojima T (2019). Production of poly(3-hydroyxybutyrate) by a novel alginolytic bacterium *Hydrogenophaga* sp. strain UMI-18 using alginate as a sole carbon source. J Biosci Bioeng.

[CR66] Zhang L, Jiang Z, Tsui TH, Loh KC, Dai Y, Tong YW (2022). A review on enhancing *Cupriavidus* necator fermentation for poly(3-hydroxybutyrate)(PHB) production from low-cost carbon sources. Front Bioeng Biotechnol.

